# Telomeres and the natural lifespan limit in humans

**DOI:** 10.18632/aging.101216

**Published:** 2017-04-06

**Authors:** Troels Steenstrup, Jeremy D. Kark, Simon Verhulst, Mikael Thinggaard, Jacob V. B. Hjelmborg, Christine Dalgård, Kirsten Ohm Kyvik, Lene Christiansen, Massimo Mangino, Timothy D. Spector, Inge Petersen, Masayuki Kimura, Athanase Benetos, Carlos Labat, Ronit Sinnreich, Shih-Jen Hwang, Daniel Levy, Steven C. Hunt, Annette L. Fitzpatrick, Wei Chen, Gerald S. Berenson, Michelangela Barbieri, Giuseppe Paolisso, Shahinaz M. Gadalla, Sharon A. Savage, Kaare Christensen, Anatoliy I. Yashin, Konstantin G. Arbeev, Abraham Aviv

**Affiliations:** 1 Epidemiology, Biostatistics and Biodemography, Institute of Public Health, University of Southern Denmark, Odense 5000, Denmark; 2 Epidemiology Unit, Hebrew University-Hadassah School of Public Health and Community Medicine, Jerusalem 91120, Israel; 3 Groningen Institute for Evolutionary Life Sciences, University of Groningen, Groningen, The Netherlands; 4 Department of Clinical Genetics, Odense University Hospital, Odense 5220, Denmark; 5 Danish Aging Research Center, University of Southern Denmark, Odense 5000, Denmark; 6 The Danish Twin Registry, University of Southern Denmark, Odense 5220, Denmark; 7 Department of Public Health, Environmental Medicine, University of Southern Denmark, 5000 Odense C, Denmark; 8 Department of Clinical Research, University of Southern Denmark and Odense Patient Data Explorative Network (OPEN), Odense University Hospital, Odense, Denmark; 9 Department of Twin Research and Genetic Epidemiology, King’s College London, London, UK; 10 NIHI Biomedical Research Center at Guy’s and St Thomas Foundation Trust, London SE1 9RT, UK; 11 Center of Human Development and Aging, Rutgers, The State University of New Jersey, New Jersey Medical School, Newark, NJ 07103, USA; 12 Department of Geriatrics, University Hospital of Nancy, F54500, France; 13 INSERM, U1116, Vandoeuvre-les-Nancy, F54500, France; 14 Université de Lorraine, Nancy, F54000, France; 15 Population Sciences Branch of the National Heart, Lung and Blood Institute, Bethesda, MD and the Framingham Heart Study, Framingham, MA 01702, USA; 16 Cardiovascular Genetics Division, Department of Medicine, Cornell University, Ithaca, NY 14850 USA; 17 Department of Epidemiology, University of Washington, Seattle, WA 98195, USA; 18 Center for Cardiovascular Health, Tulane University, New Orleans, LA 07118, USA; 19 Department of Medical, Surgery, Neurologic, Metabolic and Aging Science, University of Campania “Luigi Vanvtelli” 80138 Naples, Italy; 20 Clinical Genetics Branch, Division of Cancer Epidemiology and Genetics, National Cancer Institute, Bethesda, MD 20890, USA; 21 Biodemography of Aging Research Unit, Social Science Research Institute, Duke University, Durham, NC 27708, USA

**Keywords:** maximal lifespan, life-expectancy, longevity, sex, leukocytes

## Abstract

An ongoing debate in demography has focused on whether the human lifespan has a maximal natural limit. Taking a mechanistic perspective, and knowing that short telomeres are associated with diminished longevity, we examined whether telomere length dynamics during adult life could set a maximal natural lifespan limit. We define leukocyte telomere length of 5 kb as the ‘telomeric brink’, which denotes a high risk of imminent death. We show that a subset of adults may reach the telomeric brink within the current life expectancy and more so for a 100-year life expectancy. Thus, secular trends in life expectancy should confront a biological limit due to crossing the telomeric brink.

## Introduction

Cardiovascular disease (CVD), principally due to atherosclerosis, remains the largest cause of death and therefore influences longevity in the US [1,2]. This also applies to other middle/high-income countries. Two large meta-analyses have concluded that short leukocyte telomere length (LTL) is associated with CVD [3,4], while other studies [5–8], including a meta-analysis [9], have shown that short LTL predicts diminished longevity. Thus, the association between short LTL and diminished longevity might relate in part to the association of short LTL with CVD. Moreover, genetic analyses have revealed that alleles associated with short LTL are also associated with CVD [10–12], largely excluding the possibility of reverse causality, i.e., CVD causing LTL shortening. Coupled with observations that LTL, which reflects telomere length in somatic tissues [13], is highly heritable [14,15] and is largely determined at birth [16], these findings suggest that telomere length might play an active role in CVD and longevity. Such a conclusion is relevant to the debate about the existence of a natural lifespan limit for humans [17–21]. Here we show that in some individuals LTL becomes critically short at an age younger than that of the current life expectancy. Moreover, we show that with predicted upward trajectories of life expectancy, the proportion of these individuals will only increase in the general population.

## RESULTS

We examined the potential role of telomere length in human longevity in two settings: contemporary life expectancy, and life expectancy of 100 years (LE-100) i.e., assuming survival until the age of 100 years [17]. To this end, we first defined the LTL threshold below which the probability of survival substantially declines. We refer to this threshold as the ‘telomeric brink’ (TB).

**Figure 1:**
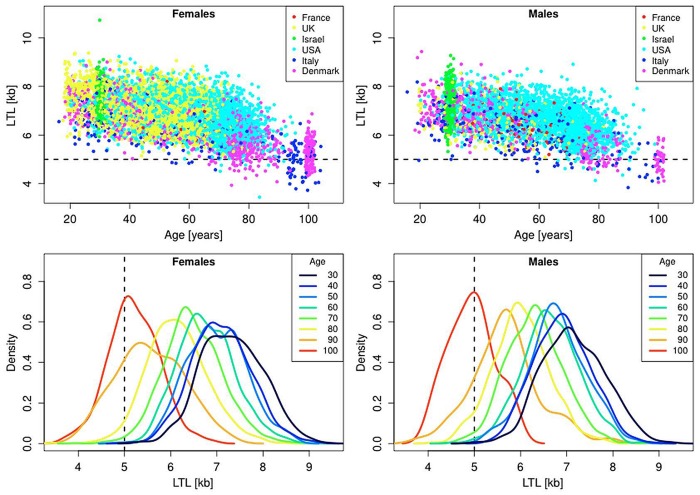
**Scatter plots and density plots of LTL as a function of age for males and females residing in different countries.** Measurements of LTL were performed in the same laboratory on DNA donated by participants in different studies in different countries (Supplemental Table 1). The horizontal dashed lines in the top panels and vertical dashed lines in the bottom panels indicate LTL values of 5 kb. The bottom plots are smoothed histograms obtained by kernel density estimation.

Figure 1 is a composite that illustrates the relation between LTL, measured in the same laboratory, and age for whites of European ancestry residing in Denmark (n=1,727), France (n=185), Italy (n=548), the UK (n=3,514), the USA (n=5,726), and Jews in Israel (n=620). Characteristics of these subjects are displayed in Table 1S. The scatter plots (Figure 1, top panels) show that LTL progressively becomes shorter with age and that it varies widely between individuals of the same age.

**Table 1:** Probability of reaching the LTL brink of 5.0 kb at ages 35, 50, 65 and 80 years for period life table mortality and for a life expectancy of 100 years by quintiles of LTL ranking, assuming that yearly LTL attrition (in bp) is independent and gamma distributed with shape parameter 0.4 and scale parameter 75.

Age (yrs)	35		50		65		80	
Life Expectancy (yrs)	Period	LE-100	Period	LE-100	Period	LE-100	Period	LE-100
All (%)	14	39	8	27	6	20	10	24
Prob Q1 (%)	50	93	34	83	25	73	45	88
Prob Q2 (%)	16	62	6	37	3	21	4	28
Prob Q3 (%)	5	30	1	12	0	5	0	5
Prob Q4 (%)	1	10	0	2	0	1	0	1
Prob Q5 (%)	0	1	0	0	0	0	0	0

LTL= leukocyte telomere length; Period = mortality based on period life tables; LE-100 = expectancy of 100 years for all; Prob = probability of reaching the telomeric brink; Q1 = 0-19% of the LTL distribution, Q2 = 20-39% of the LTL distribution, Q3 = 40-59% of the LTL distribution, Q4 = 60-79% of the LTL distribution, Q5 = 80-99% of the LTL distribution.

To establish a reference for a critically short LTL that engenders a considerable risk of imminent death, i.e., the TB, we measured LTL (in the same laboratory that generated the LTL data displayed in Figure 1) in individuals suffering from dyskeratosis congenita (DC) and their unaffected relatives. DC, the outcome of rare germline mutations, is expressed in extremely short telomere length, which is the main cause of the patients’ premature demise [22]. In patients with DC the mean LTL was 4.99 kb, while in relatives it was 6.49 kb (p<0.0001) (Table 2S).

Although DC and related telomere disorders [23] unfold under different circumstances from those experienced by the individuals with short telomeres in late life, for the following reason LTL of 5 kb is a reasonable cutoff point to define a critically short LTL: Only 0.78%, of live subjects younger than 90 years displayed LTL ≤ 5 kb (Figure 1 top panel). In the oldest old, i.e., between the ages of 95-105 years, where mortality rate is very high, 37% (95% CI: 30-44%) of females showed an LTL ≤ 5 kb, compared with 58% (95% CI: 43-72%) of males (p=0.009 by Fisher's exact test). These cross-sectional data provide no direct information about selective survival with respect to LTL. A variance-ratio test suggests, however, that this might be the case (Figure 1, bottom panels), as, for instance, individuals aged 100 (95-105) years displayed less LTL variance than those aged 50 (45-55) years (variance ratios of 1.60 for males, p = 0.048; variance ratio of 1.36 for females, p = 0.011).

Figure 1 does not offer information that foretells if a person whose LTL is, for instance, 6.4 kb at the age of 35 years could reach his/her life expectancy before crossing the TB. However, with information from life tables [24], combined with the known rate of telomere shortening with age [25], we estimated the proportion of individuals reaching the TB during their predicted life expectancy. We also calculated the proportion of individuals reaching the TB for LE-100. This estimated proportion indicates to what extent living to 100 years may be constrained by LTL.

We computed the proportion of our composite study sample that would reach the TB based on the baseline LTL for four age groups, 35 (30-40), 50 (45-55), 65 (60-70) and 80 (75-85) years, and for different LTL attrition rates (Materials and Methods). Our initial computations were strictly based on the empirical data displayed in Figure 1 and the assumption of a constant rate of age-dependent LTL attrition for a given individual. For individuals who had already crossed the TB at baseline, we let the TB age be equal to their age at blood collection. We then found the individual’s probability of reaching the TB through reference to the corresponding period life table [24], based on the nationality, sex and age of the individual and the date of blood collection. By taking the mean of the individual probabilities of reaching an LTL= 5 kb, we determined the overall probability of reaching the TB for a given LTL attrition rate. We used the same approach to quantify the probability of reaching the TB before the LE-100, except that instead of using period life table mortality, we used a lifespan of exactly 100 years. Thus, an individual is judged to have reached the TB age if his/her LTL becomes shorter than 5 kb before the age of 100 years.

**Figure 2:**
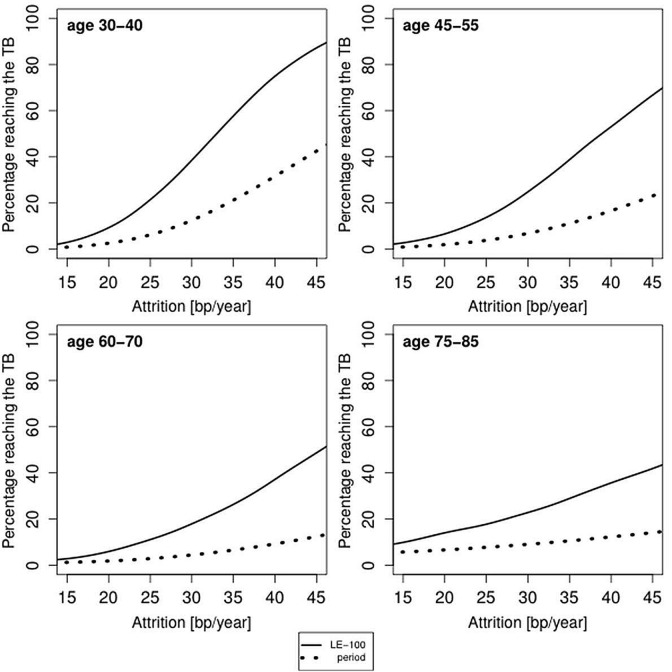
**Predicted proportion of the composite study population reaching the telomere brink (TB; 5 kb) based on period life table mortality (period), life expectancy of 100 years (LE-100) and LTL attrition.** The panels display findings for four age groups: 35 years (range 30-40 years); 50 years (range 45-55 years); 60 years (range 55-65 years); 80 years (range 75-85 years), based on different LTL attrition rates (15-45 bp/year). For period mortality, the proportion (in %) of individuals reaching an LTL of 5 kb before their life expectancy is based on the aggregate mortality data for a given country and sex at the time of blood collection.

Younger subjects (ages 35 and 50 years) in the samples displayed in Figure 1, whose LTL attrition is > 30 bp/year, already run some risk of reaching the TB based on period mortality (Figure 2). This risk escalates for LE-100, such that even individuals whose LTL attrition rates are as low as 20 bp/year could be at risk. Given that a longer life expectancy entails a greater chance of reaching the TB, the increased probability of reaching the TB based on LE-100 is expected, but what is relevant here is that this effect is of quantitative importance.

**Figure 3:**
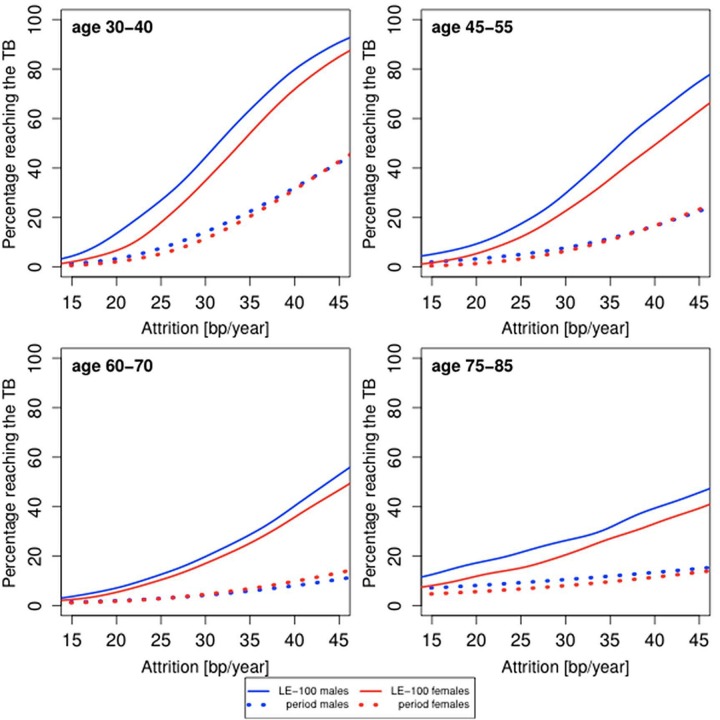
**Predicted proportion of the composite study population of males and females reaching the telomere brink (TB; 5 kb) based on period life table mortality (period), life expectancy of 100 years (LE-100), LTL ranking and LTL attrition.** The panels display findings for four age groups: 35 years (range 30-40 years); 50 years (range 45-55 years); 60 years (range 55-65 years); 80 years (range 75-85 years), based on different LTL attrition rates (15-45 bp/year).

Females typically have a longer LTL than males [26]. Females of modern societies also outlive males by approximately 5 years [24,27]. The Human Mortality Database for 2010 [24] indicates, for instance, that on the average, a Danish female dies at 81.3 years, while Danish male dies at 77.1 years; a French female dies at 84.7 years, while a French male dies at 78.0 years. Typically, females display an age-adjusted LTL that is approximately 150 bp longer than that of males (data presented in Table 1S are not adjusted for age). We examined, therefore, the impact of sex on the probability of reaching the TB (Figure 3). For period life table mortality, the probabilities for males and females of reaching the TB are almost identical. However, for the fixed LE-100, the probability of reaching the TB is higher in males than in females.

**Figure 4:**
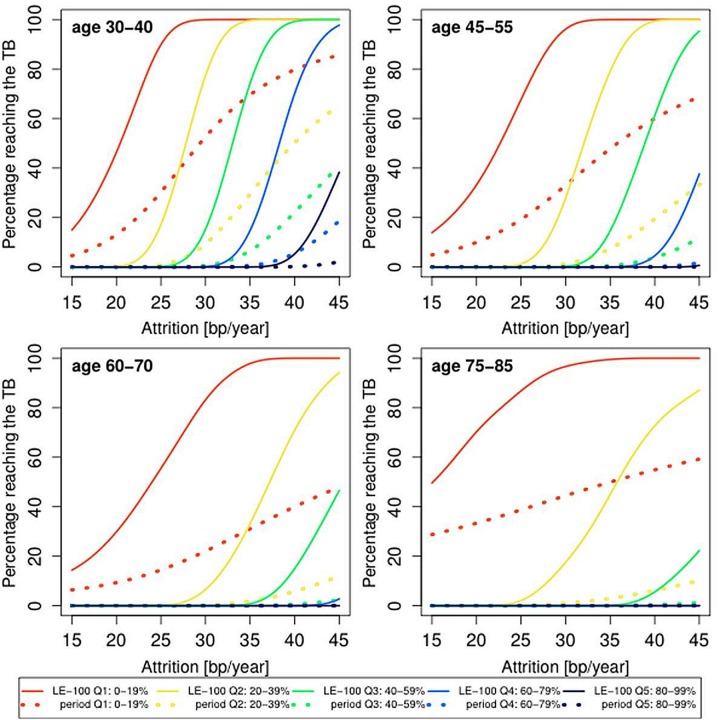
**Predicted proportion of the composite study population reaching the telomere brink (TB; 5 kb) based on period life table mortality (period), life expectancy of 100 years (LE-100), LTL ranking and LTL attrition.** Individuals were ranked by quintiles, where the shortest (1st) LTL quintile is 0-19% and the longest (5th) LTL quintile is 80-99%. The panels display findings for four age groups: 35 years (range 30-40 years); 50 years (range 45-55 years); 60 years (range 55-65 years); 80 years (range 75-85 years), based on different LTL attrition rates (15-45 bp/year).

Thus far, we presented the population average probability of reaching the TB, but this probability is likely to be heterogeneous, depending on the initial LTL. Adults typically display strong tracking of LTL, such that compared with peers, the individual’s LTL is virtually anchored to a given LTL rank at least over the course of up to 13 years of follow-up [28]. We attributed this phenomenon to the outsized influence of the inter-individual variation in LTL at birth and to a lesser extent to the inter-individual variation in LTL attrition during growth on the individual’s LTL throughout the life course [16]. We therefore examined the impact of LTL ranking by quintiles (1^st^ quintile, shortest LTL; 5^th^ quintile, longest LTL) on the probability of the individual reaching an LTL of 5 kb during his/her future life course. We observed that having an LTL ranked in the lower two quintiles of the LTL distribution significantly increased the probability of reaching the TB as compared to the higher quintiles, based on period mortality and more so based on LE-100 (Figure 4).

The probabilities displayed in Figures 2-4 underscore that a subset of the general population might reach the TB at present life expectancies and more so at LE-100. However, they are based on constant rates of LTL attrition throughout the adult life course. In reality, the individual’s rate of LTL attrition probably fluctuates. Accordingly, we have also modeled the probability of reaching the TB with a theoretical distribution for the yearly LTL attrition based on randomized, variable LTL attrition rates over a 10-year LTL shortening of 300 bp ± 150 bp (SD) [25].

Table 1 displays the aggregate findings for the probability of reaching the TB inferred for the general population and the five LTL ranking quintiles. In this setting, for instance, a 35 year-old individual has a 14% and 39% probability of reaching the TB for period mortality and LE-100, respectively. An individual ranked in the lowest (1^st^) quintile of the LTL distribution has a 50% and a 93% probability of reaching the TB for period mortality and LE-100, respectively. We note that the dataset is a composite of studies comprising individuals of different age groups and nations (Figure 1). Thus, some of the small variations in the probability of reaching the TB for “All” and specific quintiles in Table 1 might reflect different mean ages of mortality in different nations. The relevant comparisons of interest are between period mortality and LE-100 and between individuals ranked in the upper versus lower quintiles.

## DISCUSSION

Our analysis suggests that the individual’s LTL, as reflected in his/her ranking, may be a major determinant of that individual’s natural lifespan limit from the standpoint of telomere biology. Clearly, the link between LTL and human longevity is more complex, since telomere biology might also contribute to human aging and longevity by mechanisms additional to a probability of having critically short telomeres that impact cell viability and the individual’s survival. For instance, age-dependent telomere shortening might alter gene expression in sub-telomeric regions [29], thus influencing aging and longevity. Moreover, double stranded DNA breaks in telomeres are irreparable and can bring about cell senescence and affect cellular viability when TL is not critically short [30]. That said, the TB concept underscores the fact that critically short telomeres can be reached in a subset of the general population for current life expectancy and in a substantially larger proportion for the LE-100.

Demonstrating that for period life table mortality the probability of reaching the TB is similar in males and females suggests that the female life expectancy advantage is approximately equivalent in terms of telomeric attrition years to the LTL sex gap. For instance, assuming tha females live 5 years longer than males, an LTL that is longer by 150 bp in females amounts to 5 years of telomeric equivalence for an average of 30 bp/year LTL attrition rate during adulthood. Further support for this thesis is provided by the trajectories based on the fixed LE-100. As females have a longer LTL than males, their risk of reaching the TB is smaller than that of males within a 100-year lifespan. Empirical data displayed in Figure 1 support this thesis; between the ages of 95-105 years, the fraction of males who have reached the TB of 5 kb is considerably higher than that of females. Collectively, these findings point to an association between LTL and the sex difference in lifespan.

Observing a nontrivial proportion with LTL ≤ 5 kb in the oldest old but not in the general population might stem from selection on two levels. First, in the context of current life expectancy, the oldest old are naturally a highly selected group. Their TB might be situated at lower LTLs. Second, epidemiological studies in the general population rarely recruit moribund, very sick individuals, who might display LTL ≤ 5 kb. However, studies of the oldest old rarely exclude participants in poor health. Therefore, if an LTL of 5 kb denotes a high probability of death or even imminent death, it is unlikely to be found in the general adult population but more likely to be found in the oldest old.

In conclusion, at present, most individuals are not reaching the LTL brink during their life course, but our findings suggest that further extension in human longevity will be increasingly constrained by telomere length. This inference requires an assumption that a possible increase in telomere length at birth and a decrease in the average rate of telomere length attrition after birth in future generations will not offset this prediction. Notably, however, potential interventions to forestall the telomeric brink may have adverse consequences. While short LTL [3,4] and alleles associated with a shorter LTL [10–12] increase CVD risk, recent studies show that long LTL [31–34] and alleles associated with long LTL [12,35–38] increase risk of major cancers. Such findings beg the (evolutionary) question: Why is human telomere length as long as it is? Emerging data suggests that evolution has been fine-tuning our telomere length to balance cancer against degenerative diseases [39–41]. In contemporary humans, this balance has ostensibly influenced longevity beyond the reproductive years.

## MATERIALS AND METHODS

### Subjects

The LTL dataset was based on LTL measurements performed for cohorts participating in several studies [42–49]. Patients with DC and their unaffected relatives were participants in the National Cancer Institute’s Longitudinal Inherited Bone Marrow Failure Syndromes Study (NCI Protocol 02-C-0052, ClinicalTrials.gov Identifier NCT-00027274). DC patients included in this analysis had to have a germline mutation in one of the known causative DC genes and/or at least two features of the diagnostic triad (oral leukoplakia, reticular skin pigmentation, and/or nail dysplasia) along with other clinical findings consistent with DC. All relatives are mutation-negative except for 6/46 (13%); the probands in these 6 families are negative for known genes but have the diagnostic triad and other features consistent with DC.

All subjects whose LTL data are summarized in Tables S1 and S2 and those displayed in Figure 1 provided written informed consent approved by various institutional review boards and equivalent committees in the US, Europe and Israel.

### Measurements of LTL by Southern blots of the terminal restriction fragments

These measurements were performed as previously described using the restriction enzymes Hinf I/Rsa I [50].

### Modeling

For a chosen rate of attrition, we find each individual's TB age by:

$$TBage=age+\frac{LTL-5kb}{atrition}$$

where age is the age when the blood was collected. In case an individual has LTL < 5 kb, we let the TB age be the age at blood collection. The probability of survival until the TB age is determined through lookup in period life tables [24] or by assuming survival until the age of 100 years. We aggregate the individual probabilities in order to find the overall probability of reaching the TB age. To construct Figures 2-4, we have used attrition rates of 15-45 bp/year in steps of 1 bp/year. The smooth curves in Figures 2-4 were obtained by kernel smoothing of these point-estimates (normal kernel and a bandwidth of 5 bp/year).

For illustration, consider a 50 year-old French woman who had blood drawn in 1998 for measurements of LTL. If this individual’s LTL was 6.1 kb and her constant attrition is assumed to be 30 bp/year, she would reach the TB of 5 kb after 37 years (her TB age is 87 years). The Human Mortality Database for French females in 1998 shows that 46% of women, aged 50 years, live to be 87 or more. Thus, the probability for this 50 year-old French woman of reaching the brink is 46%.

We also modeled the probability of reaching the TB using a theoretical distribution of variable yearly LTL attrition. In order to find the aggregate probability of reaching the TB, we modeled the LTL attrition based on observations made in longitudinal studies with at least 10 years of follow-up, because measurement errors of LTL in longitudinal studies of shorter duration might produce an artifact in the form of LTL elongation [25]. We chose to model changes to LTL as the sum of yearly independent attrition, where the yearly attrition (in bp) is drawn from a gamma distribution with shape parameter 0.4 and scale parameter 75. The shape and scale parameters were chosen such that the total LTL attrition after 10 years has a mean of 300 bp and an SD of 150 bp . The gamma distribution was chosen (Figure S1) because it gives a rather good fit to observed data from a comprehensive longitudinal study of approximately 12 years LTL attrition, where LTL was measured by Southern blots.

This model enabled finding the theoretical LTL attrition distribution for any number of years after blood collection. When combined with mortality data, this approach allows us to effectively aggregate the curves in Figures 2 and 3 into single values for the probability of reaching the TB of 5 kb (Table 1) for each age group for the general population and for LTL ranking by quintiles.

## Supplementary Material

Supplementary File
