# Supplementary material for: Telomeres and the natural lifespan limit in humans
Source: Aging (Albany NY). 2017 Apr 6;9(4):1130–42. doi: 10.18632/aging.101216 (PMC5425118; doi:10.18632/aging.101216)
Supplement: Supplementary File [file aging-09-1130-s001.pdf]

## SUPPLEMENTARY MATERIAL

**Table 1S. Subject characteristics.**

| Country/Sex | N | Telomere Length (kb) (2.5$^{th}$ - 97.5$^{th}$ percentile) | Age (years) (2.5$^{th}$ - 97.5$^{th}$ percentile) | Period* |
|---|---|---|---|---|
| France/females | 61 | 6.5 (5.5 – 7.6) | 59 (37 – 75) | 1998 |
| France/males | 124 | 6.4 (5.3 – 7.5) | 57 (38 – 74) | 1998 |
| Denmark/females | 1159 | 6.3 (4.7 – 8.0) | 64 (22 – 102) | 1996-2005 |
| Denmark/males | 568 | 6.4 (4.7 – 8.0) | 55 (21 – 102) | 1996-2005 |
| Israel/females | 207 | 7.5 (6.5 – 8.7) | 30 (29 – 32) | 1990 |
| Israel/males | 413 | 7.3 (6.1 – 8.6) | 30 (29 – 32) | 1990 |
| USA/females | 3187 | 6.8 (5.5 – 8.2) | 62 (32 – 83) | 1992-1996 |
| USA/males | 2539 | 6.6 (5.4 – 7.9) | 62 (33 – 84) | 1992-1996 |
| Italy/females | 284 | 6.0 (4.3 – 7.7) | 60 (24 – 101) | 2004 |
| Italy/males | 264 | 6.1 (4.6 – 7.6) | 54 (23 – 96) | 2004 |
| UK/females | 3200 | 7.0 (5.8 – 8.4) | 49 (22 – 71) | 1998 |
| UK/males | 314 | 6.6 (5.4 – 8.0) | 47 (21 – 76) | 1998 |

**Table 2S. Characteristics of patients with dyskeratosis congenita and their relatives.**

| Characteristics | DC | Relatives |
|---|---|---|
| Number (male:female) | 23 (16:7) | 45 (18:27)* |
| Age (years) | | |
| Mean±SD | 30.8±15.4 | 38.4±17.2 |
| Median (range) | 30.3 (7.4-71) | 40.6 (7.7-69.4) |
| LTL (kb) | | |
| Mean±SD | 4.99±0.79 | 6.49±0.71 |
| Median | 4.66 (3.83-6.55) | 6.55 (4.51-7.86) |

DC, dyskeratosis congenita; LTL, leukocyte telomere length; *6 relatives of DC patients for whom the causative mutation is unknown displayed mean=6.96 kb, median=7.2 kb (range=5.59-7.86 kb).

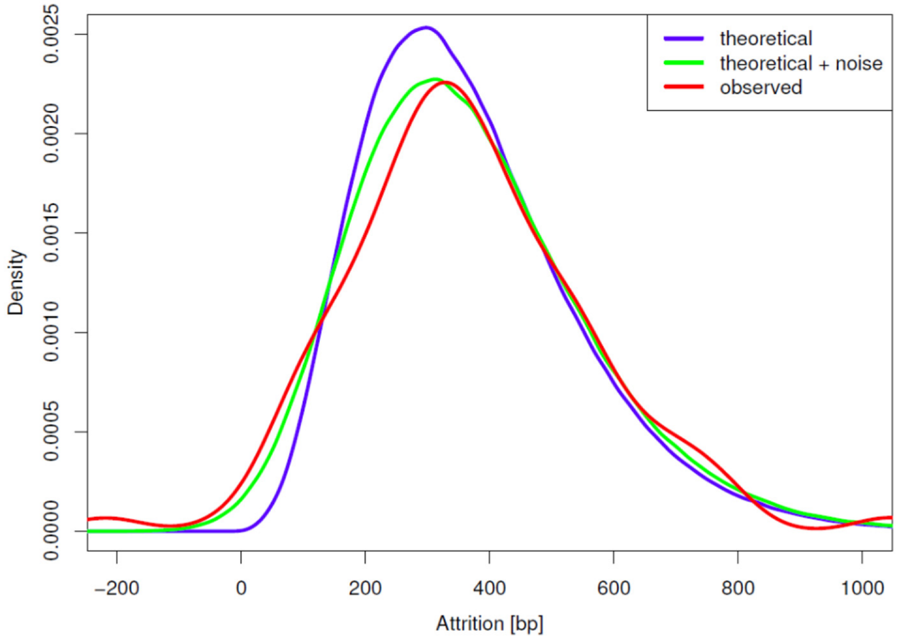


**Figure S1. Observed LTL attrition distributions after approximately 12 (5th - 95th percentile: 11.2-17.8) years along with theoretical distributions (one with- and one without measurement error).** The observed LTL attrition distribution is from whites of European descent [1]. The theoretical distribution is based on assumed yearly independent gamma distributed attrition (in bp) with shape parameter 0.4 and scale parameter 75 (these values have been chosen such that the 10-year attrition distribution has mean 300 bp and SD 150 bp). The theoretical curve is based on the actual follow-up times observed in the study [1] (each observation time is turned into 5000 simulations, from which the theoretical curve is found by kernel density estimation). The theoretical curve with noise is generated by further adding normally distributed noise with mean zero and SD 74. The choice of SD 74 is based on the estimated intra-assay SD for the study (1).

## REFERENCES

1. Chen, W, et al. Longitudinal versus cross-sectional evaluations of leukocyte telomere length dynamics: age-dependent telomere shortening is the rule. J Gerontol A Biol Sci Med Sci. 2011; 66: 312-319.
